# Anti-neutrophil cytoplasmic antibodies in rheumatoid arthritis: two case reports and review of literature

**DOI:** 10.1186/1710-1492-8-19

**Published:** 2012-12-19

**Authors:** David Spoerl, Yves-Marie Pers, Christian Jorgensen

**Affiliations:** 1 Unité d’Immuno-Rhumatologie, Hôpital Lapeyronie, 191 avenue du doyen, Gaston Giraud Montpellier, 34295, France

**Keywords:** Granulomatosis with polyangiitis, Rheumatoid arthritis, ANCA associated vasculitis, Rheumatoid vasculitis

## Abstract

**Background:**

Anti-neutrophil cytoplasmic antibodies are typically detected in anti-neutrophil cytoplasmic antibody associated vasculitis, but are also present in a number of chronic inflammatory non-vasculitic conditions like rheumatoid arthritis. Rare cases of granulomatosis with polyangiitis (formerly known as Wegener’s granulomatosis, a vasculitic disorder frequently associated with the presence of anti-neutrophil cytoplasmic antibodies) in patients with rheumatoid arthritis have been described in literature.

**Case presentation:**

We report two middle-aged female patients with rheumatoid arthritis who developed anti-neutrophil cytoplasmic antibodies and symptoms reminiscent of granulomatosis with polyangiitis. Despite the lack of antibodies specific for proteinase 3 and the absence of a classical histology, we report a probable case of granulomatosis with polyangiitis in the first patient, and consider rheumatoid vasculitis in the second patient.

**Conclusion:**

Taken together with previous reports, these cases highlight that anti-neutrophil cytoplasmic antibodies have to be evaluated very carefully in patients with rheumatoid arthritis. In this context, anti-neutrophil cytoplasmic antibodies detected by indirect immunofluorescence appear to have a low diagnostic value for granulomatosis with polyangiitis. Instead they may have prognostic value for assessing the course of rheumatoid arthritis.

## Background

Rheumatoid arthritis (RA) is a systemic inflammatory autoimmune disease characterized by chronic polyarthritis, eventually leading to joint erosion, and by the presence of various autoantibodies. The autoantigens recognized by these autoantibodies include cartilage components, chaperones, nuclear proteins, citrullinated proteins and enzymes. Anti-neutrophil cytoplasmic antibodies (ANCA) are present in a number of chronic inflammatory non-vasculitic conditions including RA and are therefore considered of low diagnostic value in this setting. In contrast, autoantibodies to proteinase 3 (PR3) are extremely useful as a diagnostic and disease activity indicator in granulomatosis with polyangiitis (GPA).

We report two patients with RA, as defined by the American College of Rheumatology (ACR) criteria, who developed ANCA and symptoms reminiscent of GPA, but displayed only ambiguous histology for GPA. In this context we discuss the value of ANCA as a diagnostic and prognostic tool.

## Case presentation

### Case presentation 1

A 53-years old female patient presented with erosive, seropositive, anti-cyclic citrullinated protein antibody (ACPA) positive RA in 1993. In 1999 she had an episode of auricular chondritis and in 2003 a pericarditis. In 2007 she presented with arthralgia, rectorrhagia and abdominal pain. Necrotizing intestinal vasculitis was diagnosed after intestinal resection. The histological findings, her history, plus the presence of chronic sinusitis and a four millimetres nodule at the left superior pulmonary lobe (confirmed by computer tomography) together with the presence of ANCA at 1:800 with perinuclear (p-ANCA) pattern was reminiscent of GPA and the patient was addressed to our clinic. The feasibility of a lung biopsy has been discussed, but because of the size of the nodule and the already known intestinal vasculitis, this option has been rejected. Despite the lack of ANCA specificity for either PR3 or myeloperoxidase (MPO) and the absence of a classical histology, a diagnosis of GPA was made and treatment with monthly intravenous 1.2g cyclophosphamide was started. After two months her arthralgia and abdominal pain improved, the pulmonary nodule resolved and treatment was discontinued after four months. In 2009 a bilateral meatomy was performed for her recurrent sinusitis. There were no signs of vasculitis and no granulomas in the collected tissues. Rituximab and abatacept showed no efficacy on her polyarthritis and the treatment was changed to tocilizumab in 2010. Since then she continues to have mild active GPA with recurrent sinusitis and chondritis but shows no other organ involvement and her RA is in remission (Table 1).

**Table 1:** Initial presentation and autoantibodies in case 1 and 2

	**Case 1**	**Case 2**
**Initial symptoms**	Arthralgia, abdominal pain, cough, facial tenderness.	Cough, arthralgia, morning stiffness, depression, sicca syndrome.
**Initial clinical findings**	Four tender and swollen finger joints (not better described), axillary lymphadenopathy left, tenderness in the left iliac fossa. No signs of neuropathy.	Tender right 3rd MCP joint and right shoulder, erythema right lower limb. No signs of neuropathy.
**Histology**	Necrotizing vasculitis in tissue from the intestinal resection. No signs of vasculitis and no granulomas in the collected sinus tissue.	Transthoracic biopsy: lymphohistiocytic infiltrate with central fibrinoid necrosis, no granulomas. Transbronchial biopsy: lymphogranulocytic infiltrate, no signs of vasculitis.
**Initial laboratory values**	CRP, ESR, cell counts and creatinine: within normal range, immunoelectrophoresis with modest hypogammaglobulinemia (IgG, IgA), proteinuria and cryoglubulins negative.	ESR 24mm, leucocytes 3000/mm3, CRP 20mg/l (<5), creatinine, C3, C4 within normal range, normal immunoelectrophoresis, Anticardiolipin antibodies and cryoglubulins negative, urine analysis without proteinuria.
**Autoantibodies when first tested in our clinic**	ANA 1:160, Anti-dsDNA negative, Anti-ENA negative, RF negative, ACPA 99U/ml (<20), ANCA 1:800, Anti-PR3 negative, Anti-MPO negative.	ANA 1:320, Anti-dsDNA 75U/ml (<55), Anti-ENA negative, RF 63U/ml (<15), ACPA negative, ANCA 1:500, Anti-PR3 negative, Anti-MPO negative, lupus anticoagulant antibodies negative.
**Initial investigations**	Abdominal scan: absence of lymphadenopathy, no signs of diverticulitis.	Radiography: normal skeletal imaging of hands and feet, normal chest radiography.
	Chest scan: 4mm nodule superior lobe left.	Schirmer Test negative.
	Sinus scan: signs of chronic maxillary bilateral sinusitis.	Salivary gland biopsy: modest lymphocytic infiltration (Focus score <1).
		Lung function: signs of obstruction.
		Computer tomography: four pulmonary nodules.
**Treatments**	Prior to 2007: Gold salt, Mycophenolate mofetil, Etanercept, MTX.	2000: Leflunomide, later MTX
	4/2007: Cyclophosphamide	2001-2002: Azathioprine.
	8/2007: Etanercept, later Adalimumab.	2/2002-8/2002: Cyclophosphamide.
	8/2008: Rituximab, MTX.	8/2002: Infliximab, MTX.
	1/2010: Abatacept, MTX.	4/2003: Etanercept, MTX.
	4/2010: Tocilizumab, MTX.	10/2004: Rituximab.

*MCP*: metacarpophalangeal, *CRP*: C-reactive protein, *ESR*: erythrocyte sedimentation rate *ANA*: antinuclear antibody (Titre), *dsDNA*: double-stranded DNA, *ENA*: Extractable nuclear antigen, *ANCA*: anti-neutrophil cytoplasmic antibodies (Titre), *PR3*: proteinase 3, *MPO*: myeloperoxidase, *ACPA*: Anti-cyclic citrullinated protein antibody, *RF*: rheumatoid factor, *MTX*: Methotrexate, *Ig*: Immunoglobulin.

### Case presentation 2

A 40-years old female patient presented with new onset of inflammatory, unclassified polyarthritis in 2000. Rheumatoid factor (RF) was positive, ACPA negative. In 2001 computer tomography showed four pulmonary nodules. Transbronchial biopsy revealed a lymphogranulocytic infiltrate, without signs of vasculitis. Transthoracic biopsy of a subpleural nodule showed a lymphohistiocytic infiltrate with central fibrinoid necrosis, but no granuloma. ANCA with cytoplasmic (c-ANCA) pattern, but no antigen specificity, were increased at 1:500. A diagnosis of GPA was made irrespective of the absence of upper airway or renal disease. Azathioprine and later cyclophosphamide treatments were introduced to control GPA with radiological (resolution of pulmonary nodules) but no clinical (arthralgia) efficacy. Infliximab allowed eventual reduction of concomitant corticosteroid treatment. In 2003 she developed ACPA and since then was considered to have RA with a concomitant history of GPA. Treatment with etanercept showed no benefit while rituximab, started in 2004 and continued twice yearly, led to long lasting remission of RA and quiescent GPA (Table 1).

## Discussion

Eleven cases of GPA in patients with RA have been reported in literature since 1970, four of which displayed ANCA specific for PR3 [1-8]. Four patients with ANCA associated vasculitis (AAV) fulfilling the ACR criteria for RA were described in 2010, but because no destructive arthritis could be detected radiologically they were not considered to have concomitant RA [9].

It has been suggested that both RA and GPA may arise from a similar genetic predisposition. The PTPN22 620W functional polymorphism has been implicated in the pathogenesis of GPA and RA [10]. More recently, a meta-analysis yielded a statistically significant association between GPA and the rs3087243 gene polymorphism in Cytotoxic T-Lymphocyte Antigen 4 (CTLA-4) [11], also known to enhance the development of ACPA-positive RA as compared with ACPA-negative RA [12].

RA is complicated by rheumatoid vasculitis (RV) in approximately 1-5% of patients. RV is a systemic necrotizing vasculitis affecting small and medium-sized vessels. RV commonly presents in seropositive patients with multiple erosions and a long lasting history of RA. It is characterized histopathologically in the acute stages by fibrinoid necrosis with an inflammatory infiltrate, and in chronic cases by vessel wall fibrosis, occlusion and recanalization. Clinical features of RV include Raynaud's phenomenon, digital infarcts, skin ulceration and mononeuritis multiplex. Cutaneous vasculitis, multifocal neuropathy, and depressed complement levels are all indicators of poor prognosis [13]. Clinically apparent RV of the gastrointestinal tract is rare, but often catastrophic, resulting in ischemic ulcers and bowel infarction [14]. In addition to RA duration, other risk factors for RV include the presence of extra-articular manifestations of RA, specifically rheumatoid nodulosis, scleritis, amyloidosis, and the presence of RF and ACPA [15]. Both RV and GPA can present with symmetrical peripheral polyarthritis. Gastrointestinal involvement occurs in approximately 1-10% in both diseases [16]. Other authors report an incidence of up to 38% in RV patients [14].

ANCA are not included in the ACR classification criteria or Chapel Hill Consensus Conference (CHCC) definitions for AAV, but their testing in the appropriate clinical context is recommended [17,18]. There are 2 main techniques used to test for ANCA, indirect immunofluorescence (IIF) and enzyme- linked immunosorbent assay (ELISA). IIF can demonstrate a perinuclear, a cytoplasmic or an atypical staining pattern [19]. These patterns reflect the staining of antigenic proteins, but are not antigen specific. While c-ANCA pattern is frequently due to a specific serine PR3, p-ANCA pattern can result from the presence of a number of different minor antigens. Positive ANCA test results have been reported in a number of rheumatic conditions, including RA, and high titres of antinuclear antibodies (ANA) can be confused for a p-ANCA, especially in systemic lupus erythematosus where high titres of these antibodies are found [19,20]. The use of cell fixatives other than ethanol, e.g., formalin and methanol, has been reported to allow differentiation of ANCA and ANA in these cases [21].

Studies of RA cohorts showed that around 16–52% of patients were ANCA positive in IIF and 3–32% in ELISA assays [9]. Up to 21 to 36% of RA patients may have an associated p-ANCA or atypical ANCA pattern in IIF and up to 10% of patients have anti-MPO antibodies as demonstrated by ELISA testing [22,23]. In a study investigating sera from RA patients, 49% had p-ANCA in IIF. Among these patients, 91% showed a specific immunostaining upon examination at higher magnification that did not correspond to "true" ANCA but to antibodies directed against nuclear or perinuclear antigenic constituents of the neutrophils [24]. In RV, up to 48% of patients show positive p-ANCA, a few (4-11%) being positive for anti-PR3 or anti-MPO [25].

In a multi-centre study, IIF had a sensitivity of 85% and a specificity of 76% for detecting GPA. The relatively low specificity was mainly due to positive p-ANCA in disease controls that included 7 patients with RV (one had positive p-ANCA) [26]. Conversely, the sensitivity of anti-PR3 antibodies for patients with GPA was 65 to 67%. This sensitivity was similar in patients with classical histology (granulomas) as compared to those with clinical characteristics of GPA, but who only displayed histological evidence of vasculitis or crescentic nephritis without granulomas. In this study, the specificity of anti-PR3 antibodies alone for “idiopathic systemic vasculitis” (mainly AAV) was 86-89% towards disease controls. The specificity was improved to 99% if anti-PR3 antibodies were found in combination with c-ANCA [26].

In a Japanese cohort, 16% of 125 RA patients were positive for ANCA against minor antigens of unknown significance. In this study, ANCA positive patients had a higher joint score and an elevated erythrocyte sedimentation rate (ESR), compared to those who were ANCA negative, but signs of vasculitis were not more frequent [27]. Other authors found that these ANCA-ACPA double positive RA patients may more frequently present with lung nodules and have erosive lesions of greater severity compared to ANCA negative RA patients, but it is uncertain if they are consequently more prone to develop RV [1,9,24]. Moreover, p-ANCA positivity in RA has been found to be an independent predictor of RA‐associated nephropathy [23] and, in early RA, predicted rapid radiographic joint destruction [28]. Other groups found no significant association between ANCA positivity and clinical, biological, and radiological disease activity assessments in RA [29]. Rather, ANCA positivity appeared to be associated with previous intake of sulphasalazine [30].

Finally, while RF can be detected in up to 37–50% of AAV patients without any prognostic value, ACPA develop in 1-2% of AAV patients. AAV patients with ACPA seem to have more severe vasculitis manifestations and unusual remittent joint manifestations, but do not develop erosive lesions, therefore excluding a simple overlap with RA [9].

## Conclusions

IIF in our patients has been performed in ethanol fixed cells, but because ANA were found only in low titres compared to ANCA (especially during follow up), false positive results are unlikely. Retrospectively, case 1 had erosive RA, showed histological evidence of vasculitis and clinical findings were indeed suggestive of concomitant GPA, but RV cannot be excluded. Auricular chondritis was suggestive for relapsing polychondritis, but the patient did not fulfil the diagnostic criteria for this disease [31].

ACPA are highly specific for RA [32]. Case 2 developed ACPA only three years after the onset of polyarthritis. While about 40% of RA patients develop these antibodies before the onset of articular symptoms, some patients become positive only throughout the course of their disease and some will remain ACPA negative [32,33]. Case 2 probably only had ACPA positive RA with pulmonary nodules, but GPA and RV are possible alternative diagnosis. RV appears less probable because of the short history of RA and the lack of erosions in this patient.

These two cases highlight the difficulty in differentiating extra-articular RA, RV and GPA in RA patients who present with signs of AAV. Most biopsies from the upper respiratory tract and the gastrointestinal tract do not show classical granulomas in GPA [16,34] and cannot confirm this diagnosis. Testing for ANCA may be helpful in discriminating RV and GPA, but results have to be evaluated with caution in RA. Positive ANCA specific for PR3 or MPO in ELISA have a high specificity for AAV, and therefore a high diagnostic value. However, because GPA in RA is rare, the corresponding positive predictive value of ANCA in this setting remains low.

In contrast, ANCA positivity in IIF has been shown to have a prognostic value for the course of early RA, by predicting a rapid joint destruction and a higher inflammatory activity, but has a low diagnostic value. Only one of our patients developed erosive disease, but both had a high inflammatory activity, demonstrated by the different drugs that were needed in order to achieve disease remission.

### Consent

Written informed consent was obtained from the patients for publication of these Case reports. A copy of the written consent is available for review by the Editor-in-Chief of this journal.

## Abbreviations

RA: Rheumatoid arthritis; RV: Rheumatoid vasculitis; ANCA: Anti-neutrophil cytoplasmic antibodies; PR3: Proteinase 3; MPO: Myeloperoxidase; GPA: Granulomatosis with polyangiitis; IIF: Indirect immunofluorescence; ELISA: Enzyme-linked immunosorbent assay; AAV: ANCA associated vasculitis; ACPA: Anti-cyclic citrullinated protein antibody; P-ANCA: ANCA with perinuclear pattern; C-ANCA: ANCA with cytoplasmic pattern; ANA: Antinuclear antibody; DsDNA: Double-stranded DNA; ENA: Extractable nuclear antigen; RF: Rheumatoid factor; MCP: Metacarpophalangeal; CRP: C-reactive Protein; ESR: Erythrocyte sedimentation rate; MTX: Methotrexate; CTLA-4: Cytotoxic T-Lymphocyte Antigen 4; Ig: Immunoglobulin.

## Competing interests

The authors declare that they have no competing interests.

## Authors’ contributions

DS was involved in the medical care of patient 1, retrospectively studied the two cases and drafted the manuscript. YMP and CJ were involved in the medical care of the two patients and helped to draft the manuscript. All authors read and approved the final manuscript.
